# Diagnostic interobserver variability of atypia assessment in columnar cell lesions among a group of expert breast pathologists in the United Kingdom and the Republic of Ireland, on behalf of the UK national coordinating committee for breast pathology

**DOI:** 10.1111/his.15402

**Published:** 2024-12-20

**Authors:** Soha El Sheikh, Mohamed A Mansour, Elena Provenzano, Abeer Shaaban, Andrew Lee, Yasmeen Mir, Rebecca McMillan‐Slater, Pauline Carder, Silvana Di Palma, Clinton Boyd, Purnima Makhija, Madhuri Warren, Susan Pritchard, Rahul Deb, Ian Ellis, Emad Rakha, Cecily Quinn, Sarah Pinder

**Affiliations:** ^1^ Research Department of Pathology University College London (UCL) Cancer Institute London UK; ^2^ Department of Histopathology Royal Free London Foundation Trust London UK; ^3^ Addenbrookes Hospital and NIHR Cambridge Biomedical Research Centre Cambridge UK; ^4^ Department of Histopathology Addenbrookes Hospital Cambridge UK; ^5^ Institute of Cancer and Genomic Sciences University of Birmingham Birmingham UK; ^6^ Cellular Pathology Queen Elizabeth Hospital Birmingham Birmingham UK; ^7^ Histopathology Department Nottingham University Hospitals NHS Trust, City Hospital Campus Nottingham UK; ^8^ Pathology Liverpool University Hospitals Foundation Trust Liverpool UK; ^9^ Department of Cellular Pathology St James's University Hospital Leeds UK; ^10^ Bradford Teaching Hospitals NHS Trust, Duckworth Lane Bradford UK; ^11^ Cellular Pathology Department Royal Surrey Hospital NHS Foundation Trust Guildford UK; ^12^ Histopathology Belfast Health and Social Care Trust Belfast UK; ^13^ Pathology Barts Health NHS Trust London UK; ^14^ Pathology Wythenshawe Hospital Manchester Foundation Trust Manchester UK; ^15^ Cellular Pathology University Hospitals of Derby and Burton NHS Foundation Trust Derby UK; ^16^ Academic Unit for Translational Medical Sciences, School of Medicine University of Nottingham Nottingham UK; ^17^ Department of Histopathology St Vincent's University Hospital Dublin Ireland; ^18^ University College Dublin School of Medicine Dublin Ireland; ^19^ School of Cancer and Pharmaceutical Sciences Kings College London London UK; ^20^ Department of Cellular Pathology Guy's and St Thomas’ NHS Foundation Trust London UK

**Keywords:** atypical intraductal epithelial proliferation, B3 lesion, columnar cell lesions, flat epithelial atypia, interobserver variation, lesions of uncertain malignant potential

## Abstract

**Aims:**

Atypical ductal hyperplasia and flat epithelial atypia (FEA) have defined diagnostic criteria, yet there is variation in the interpretation of these criteria, particularly when the atypia is present in a background of columnar cell lesions (CCLs). This study focuses upon cases which are especially challenging or difficult to classify reproducibly according to existing criteria.

**Methods and results:**

Thirteen breast pathology experts were asked to classify 10 challenging cases with CLLs as atypical or non‐atypical. Interobserver agreement was calculated. After two consensus meetings, which explored the morphological features underlying the decision, the cases were reassessed. Finally, a photomontage was compiled as a visual aid for practising pathologists representing a range of straightforward cases and others where subjective interpretation causes disagreement within current diagnostic criteria. Overall interobserver agreement and pairwise pathologist agreement coefficients were both in the fair range (*κ* = 0.22 and *κ* = 0.3–0.4, respectively). This improved to moderate or substantial agreement (*κ* = 0.6–0.8) after two consensus meetings. The most controversial cases were atypical cases that lacked the regular rounded nuclei of FEA, and non‐atypical cases that had florid architectural changes bordering on architectural atypia.

**Conclusion:**

Among expert breast pathologists, interobserver agreement in the diagnosis of atypia in CCLs was higher in cases with classical features of FEA. Consensus was difficult to achieve if nuclear or architectural atypia fell outside the classical definition of FEA, suggesting that this category does not encompass the range of low‐grade cytological atypia in CLLs. This study provides rationale for expanding the definition of atypia in CCLs other than FEA.

## Graphical abstract

Interobserver agreement for the presence of atypia in columnar cell lesions is poor, even among breast pathology experts. This can be improved through consensus meetings, but a subset of cases defy classification, because the current definition of flat epithelial atypia does not encompass the range of cytological and architectural atypia observed in these lesions.

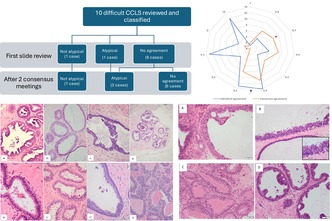

**Abbreviations**
AC: agreement coefficient
ADH: atypical ductal hyperplasia
AIDEP: atypical ductal hyperplasia
CCC: columnar cell change
CCH: columnar cell hyperplasia
CCL(s): columnar cell lesion(s)
DCIS: ductal carcinoma in situ
FEA: flat epithelial atypia
H&E: hematoxylin and eosin
IHC: immunohistochemistry
MCC: mammographic microcalcification
NCCBP: National Co‐ordinating Committee for Breast Pathology
NHSBSP: National Health Service Breast Screening Programme
Pe: chance‐agreement probability
TDLU: terminal duct lobular unit
VAB: vacuum assisted biopsy
VAE: vacuum assisted excision
WSI: whole slide image

## Introduction

The incidence of atypical lesions, which represent a grey zone in breast pathology, has substantially increased since the introduction of digital screening mammography,^1^ and the range of morphological changes to which the term atypia is being applied is wide. Atypical ductal hyperplasia (ADH), alternatively labelled atypical intraductal epithelial proliferation (AIDEP), in a core needle biopsy^2^ is seen in up to 15% of biopsies sampled for calcification.^3^, ^4^

ADH/AIDEP is characterised by partial involvement of one or two ducts by low nuclear grade cells with complex atypical growth patterns including solid, cribriform or micropapillary architectures. Flat epithelial atypia (FEA) is defined as a proliferation composed of one to several layers of cells which lack polarity and display low‐grade cytological atypia.^5^ FEA is not necessarily entirely flat, but lacks complex architecture seen in ADH and low‐grade ductal carcinoma *in‐situ* (DCIS), from which it needs to be differentiated. FEA also needs to be distinguished from columnar cell change (CCC) and columnar cell hyperplasia (CCH), which lack cytological atypia. While the distinction between ADH and epithelial hyperplasia can be improved with immunohistochemistry (IHC), the identification of atypia in the background of CCC is based only on morphology, as all these lesions have the same immunoprofile (oestrogen receptor positive and basal cytokeratin negative pattern).^6^

Distinguishing between CCC, CCH and FEA is important, and has implications for patients and the healthcare system. As per current UK NHS Breast Screening Programme (NHSBSP) guidelines,^7^ women with a diagnosis of CCC and CCH are discharged and referred back to routine screening (if pathological and radiological findings are concordant). In patients with FEA, a subsequent biopsy procedure is performed (either a vacuum‐assisted or a diagnostic surgical excision), and if not upgraded to malignancy the patient is recommended to have annual mammography for 5 years.

Diagnostic difficulties and interobserver variation in breast pathology exist and have been consistently demonstrated in atypical breast lesions.^8^, ^9^, ^10^, ^11^, ^12^, ^13^, ^14^ Low interpretive agreement on the same case by the same individual pathologist at two different time‐points was demonstrated in one study.^15^

The diagnosis of FEA has been similarly shown to be problematic, with a low concordance rate demonstrated in several studies,^16^, ^17^, ^18^, ^19^, ^20^ but none of these studies has specifically addressed the reasons behind the low concordance or the morphological appearances that make diagnostic agreement difficult to reach.

To ensure that patients are treated in a consistent manner, it is critical that we maintain the highest level of concordance in the diagnosis of atypia between pathologists in different institutions. In this study, members of the National Co‐ordinating Committee for Breast Pathology (NCCBP) investigate the concordance diagnostic rates in 10 challenging cases of breast atypia that were preselected to reflect difficult or controversial cases. The cases not only show cytological atypia but address another challenge in the assessment of FEA, which is when it is associated with focal architectural atypia, and the diagnosis of ADH may be considered. We document specific areas of difficulty and features associated with diagnostic agreement/disagreement to highlight the most diagnostically helpful features that may reduce overall interobserver variation. Lastly, we provide a validated series of images that can serve as a reference or diagnostic aid for breast pathologists when tackling FEA.

## Methods

To test consistency of opinion among a panel of breast pathology experts on the question of atypia, participants from the National Coordinating. Committee for Breast Pathology (NCCBP) reviewed 10 breast screening vacuum‐assisted biopsies (VAB) originally reported as B3 FEA but were considered difficult, and had failed to achieve consensus on local group review.^20^ The 10 cases fulfilled the following criteria:

1. Screening biopsies of mammographic microcalcification (MCC R3/R4), not masses or breast densities.
2. No concurrent or previous breast malignancy and no family risk of breast cancer.
3. No other B3 lesion present (papillary lesion, radial scar, lobular neoplasia, etc.).
4. The subsequent vacuum‐assisted excision (VAE) was benign with no residual atypia or upgrade to malignancy.
5. The subsequent first follow‐up annual mammogram was normal.

The influence of potential confounders on agreement among pathologists, such as experience or time dedicated to breast reporting and reporting practice (glass slide versus digital), were not considered.

A single whole‐slide image (WSI) from each case was scanned using the Leica Aperio Imagescope at 20× magnification. The digital slides were reviewed by 13 members of the NCCBP using the Leeds Virtual Pathology Website Slide Viewer (https://t.ly/WCJ‐).^21^ This was followed by two online consensus meetings to rediscuss the cases, and the authors analysed the morphological features of each case.

Subsequently, to focus upon the areas of difficulty identified in the first part of this study, photomicrographs of a variety of CCLs are provided. These are presented here after having undergone three rounds of discussion to classify them as atypical or non‐atypical, and a detailed description is provided highlighting the reasons behind the diagnostic decision.

### Statistical analysis

Agreement measures are reported as percentage agreement with associated standard error for each case and for the whole set of cases. We assumed the chance‐agreement probability (Pe) = 0 (best‐case scenario) and estimated the minimal required sample size (*n* = 10) to detect a statistically significant agreement coefficient of substantial agreement (*κ* = 0.65) with 90% power, as described previously.^22^ We used Gwet's agreement coefficient first‐order AC (AC1 for binary variables),^23^ together with Fleiss's kappa.^24^ Gwet's AC1 and Krippendorff's alpha (*α*) were employed to analyse responses obtained in the consensus meetings (incomplete data sets). The strength of agreement was categorised according to Gwet's AC1 as follows: *κ* = < 0.2 = poor; *κ* = 0.21–0.4 = fair; *κ* = 0.41–0.6 = moderate; *κ* = 0.61–0.8 = substantial; and *κ* = 0.81–1.0 = almost perfect.^25^ Negative values imply disagreement beyond the independence of raters or chance. All statistical analyses were performed using IBM SPSS Statistics for Windows, version 26.0 (IBM Corporation, Armonk, NY, USA).

## Results

On the digital slide review of 10 difficult cases of CCLs with atypia (https://t.ly/WCJ‐), the overall agreement was 64.1% [95% confidence interval (CI) = 49.0–79.2%]. Fleiss's kappa inter‐rater reliability value was *κ* = 0.22 (95% CI = 0.162–0.607) and Gwet's AC1 = 0.33 (95% CI = 0.031–0.64), both in the fair agreement range (Table 1).

**Table 1:** The presence or absence of atypia on digital whole slide image review of 10 difficult atypia cases. The first set of data was obtained after individual slide review and before the consensus meetings and discussion (13 participants). The second set of data was obtained after two consensus meetings and detailed discussion of the same cases (11 and nine participants). The percentage agreement (SE = standard of error) are shown and links to the digital slides used in the review are provided

Case	Links	Opinion before consensus meeting			Opinion after consensus meeting		
		Atypia present	Atypia absent	Percentage agreement (SE)	Atypia present	Atypia absent	Percentage agreement (SE)
1	476489	8	5	61.53 (3.8)	8	3	56.4 (3.5)
2	476490	11	2	84.6 (2.8)	9	2	67.3 (3.1)
3	476491	0	13	100 (0)	0	11	100
4	476492	7	6	53.84 (3.9)	3	6	56.4 (3.6)
5	476493	9	4	69.23 (3.6)	11	0	100
6	476494	13	0	100 (0)	11	0	100
7	476495	10	3	76.92 (3.3)	3	6	61.1 (3.6)
8	476497	8	5	61.53 (3.8)	7	2	61.1 (3.3)
9	476498	11	2	84.61 (2.8)	11	0	100
10	476533	7	6	53.84 (3.9)	5	4	44.4 (4.0)

Pairwise comparison between individual pathologists using Gwet's AC ranged from disagreement (−0.38) to perfect agreement^1^ (Table 2). Some pathologists had higher levels of agreement with other participants, while one pathologist disagreed with most other participants.

**Table 2:** Comparison of consensus agreements among participating pathologists in a pairwise manner in the overall assessment of the cases using Gwet's AC1. The following scale was used for interpretation: *κ* = < 0.2 = poor; *κ* = 0.21–0.4 = fair; *κ* = 0.41–0.6 = moderate; *κ* = 0.61–0.8 = substantial; and *κ* = 0.81–1.0 = almost perfect. Some negative values were obtained indicating disagreement beyond that expected by chance

	P1	P2	P3	P4	P5	P6	P7	P8	P9	P10	P11	P12
P2	0.31											
P3	0.655	0.706										
P4	0.231	0.31	0.31									
P5	0.406	0.083	0.45	0.01								
P6	0.406	0.083	0.45	0.01	0.2							
P7	−0.2	0.231	−0.154	0.2	−0.386	0.406						
P8	0.655	0.706	0.706	0.31	0.083	0.45	0.231					
P9	0.31	1	0.706	0.31	0.083	0.083	0.231	0.706				
P10	0.406	0.45	0.083	0.01	0.2	−0.2	0.01	0.45	0.45			
P11	0.52	0.866	0.866	0.52	0.31	0.31	0.083	0.866	0.866	0.31		
P12	0.615	0.31	0.655	0.231	0.802	0.406	−0.2	0.31	0.31	0.01	0.52	
P13	0.406	0.083	0.45	0.406	0.6	0.6	0.01	0.083	0.083	−0.2	0.31	0.802

Subsequently, two consensus meetings were organised that allowed participants to discuss the cases and analyse their diagnostic features (Table 3), then interobserver agreement was re‐assessed. This increased from 64.1 to 77.2% (95% CI = 61.0–93.1%), *κ* = 0.6 (95% CI = 0.27–0.92) and *α* = 0.484 (95% CI = 0.01–0.89), indicating moderate agreement after the two consensus meetings (Table 1).

**Table 3:** Summary of the consensus expert panel discussions of the 10 cases and the most salient features advocated for atypia. Histologically, architectural atypia in the form of micropapillae was noted in cases 2, 8, 9 and 10, while those that contained bridges/solid growth included cases 1, 4, 9, 10. During the discussions, the participants agreed that low cytological atypia (if present) in all cases except cases 1, 2, 4 and 7, where comments have been made that atypia is beyond low grade, and is more akin to intermediate grade nuclear size. No cases with high nuclear grade were present

	Case 1	Case 2	Case 3	Case 4	Case 5	Case 6	Case 7	Case 8	Case 9	Case 10
TDLU										
Dilation	No	Yes (lobulocentric)	Yes	Yes (mainly ducts)	Yes	Yes	Yes	Yes	Yes	Yes
Contour	Irregular	Mixed*	Mixed*	Mixed*	Mixed*	Irregular	Irregular	Mixed*	Rounded	Rounded
Nuclei										
Shape	Round	Round	Round and oval	Irregular	Round and oval	Round	Irregular	Round and oval	Round and oval	Rounded and oval
Polarity	Lost	Lost	Maintained	Lost	Lost	Lost	Maintained	Lost	Lost	Lost in areas
Nucleoli	Tiny	Tiny	Not visible	Not visible	Tiny	Tiny occasional	Not visible	Occasional pinpoint	Not visible	Not visible
Equivalent nuclear size	Low to intermediate	Intermediate	Low	Intermediate	Low	Low	Intermediate	Low	Low	Low
Monotony	No	No	No	No	No	Yes	No			
Architecture^†^	Complex	Complex	Simple	Complex	Simple	Complex	Simple	Simple	Complex	Complex
Apical snouts	focal	Present	Present	Present	Present	Absent (mucin)	Present	Present	Present	Present
Extent	Multifocal	Multifocal	NA	Multifocal	Unifocal	Unifocal	Multifocal	Unifocal	Multifocal	Unifocal
Size of largest focus	1.5 mm	1.1 mm	NA			2.3 mm	1.2		2.5 mm	2.1 mm
Mitoses	Absent	Present	Absent	Present	Absent	Absent	Absent	Absent	Absent	Absent
Associated inflammation	Mild	Mild	Absent	Absent	Mild	Mild	Moderate	Mild	Mild	Mild
IHC recommended	Yes, to define larger cells	No	No	No	No	No	No	No	No	No

* Architectural changes were described as simple if they included stratification, tufts and mounds, and as complex if there were bars, bridges and micropapillae. TDLU, terminal duct lobular unit; NA, not available; IHC, immunohistochemistry.

^†^ Mixed contours refers to the presence of both rounded and irregular glandular outlines.

**Figure 1:**
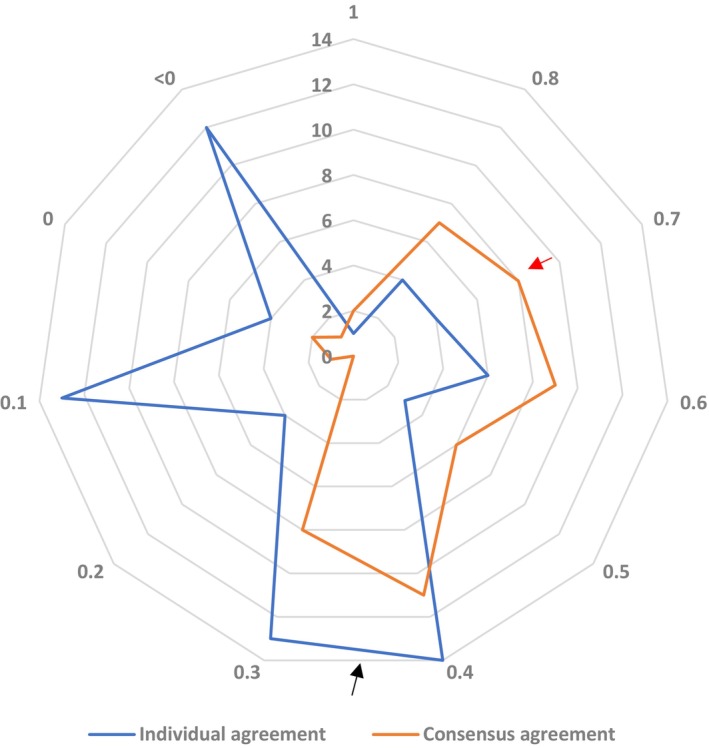
Pairwise comparison of pathologists’ agreement. On individual review prior to the consensus meetings, the highest frequency of pairwise agreements was in the fair agreement range (*κ* = 0.21–0.4; black arrow), followed by a second peak in the poor agreement range (*κ* = 0–0.2) with some negative correlations suggesting disagreement. In contrast, substantial or moderate agreement (*κ* = 0.6–0.8; red arrow) was more frequent after two consensus meetings and review, overall shifting to the right (*κ* = < 0.2 = poor; *κ* = 0.21–0.4 = fair; *κ* = 0.41–0.6 = moderate; *κ* = 0.61–0.8 = substantial; and *κ* = 0.81–1.0 = almost perfect).

When agreement coefficients before and after the consensus meetings were compared, both were most frequently in the fair agreement range (*κ* = 0.3–0.4) (Figure 1). A second peak was observed in the poor agreement range (*κ* = < 0.2) on individual review that shifted to the right after the consensus meetings, together with the peak in the moderate agreement range, indicating improved diagnostic agreement of FEA/AIDEP on group review.

### Specific comments relating to case review

#### Group 1 cases (achieving 100% agreement)

**Figure 2:**
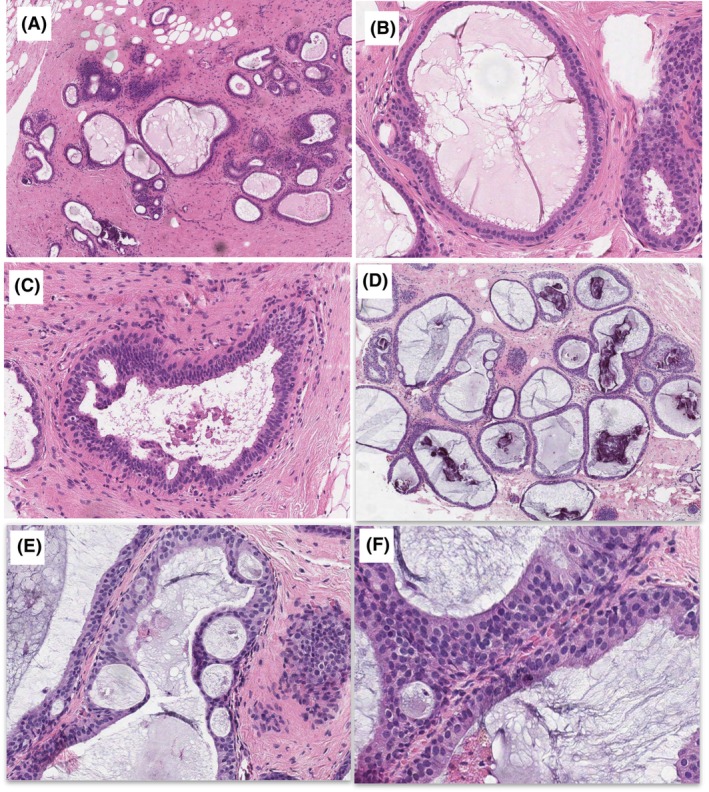
Group 1 cases with perfect agreement among participants. Case 3 (**A–C**), 100% agreement non‐atypical) had a mixed architecture with mainly irregular luminal outlines (**A**). The ducts were lined by fairly uniform round basal nuclei with stratification, some nuclear enlargement and loss of polarity in adjacent lobules. **B**, A single duct contained rigid spaces (**C**), early bridges and bars, not the cells that lacked polarisation towards the space (not Roman bridges). In contrast, case 6 (**D–F**, 100% agreement atypical), had a uniformly rigid architecture with mucin and microcalcification over an area of 2.3 mm (**D**). The focal rigid punched‐out spaces were surrounded by streaming and thinning bridges, not Roman bridges, as classically described (**E**). The nuclei were rounded and uniform with loss of polarity where stratified (**F**).

The most significant features to indicate benignity in case 3 were the small, basally situated elongated nuclei, arranged perpendicularly to the basement membrane with preserved polarity. In terms of architecture, there were tapering bridges and streaming, which were considered reassuring for CCH rather than FEA or ADH (Figure 2A–C). In contrast, in case 6, the most significant features of FEA/AIDEP were the uniform low‐grade monotonous rounded nuclei with loss of polarity and occasional micronucleoli (Figure 2D–F). Focal architectural atypia in the form of bridges, but no true micropapillae, rigid bars, cribriform architecture were present.

#### Group 2 cases (achieving > 70% agreement)

**Figure 3:**
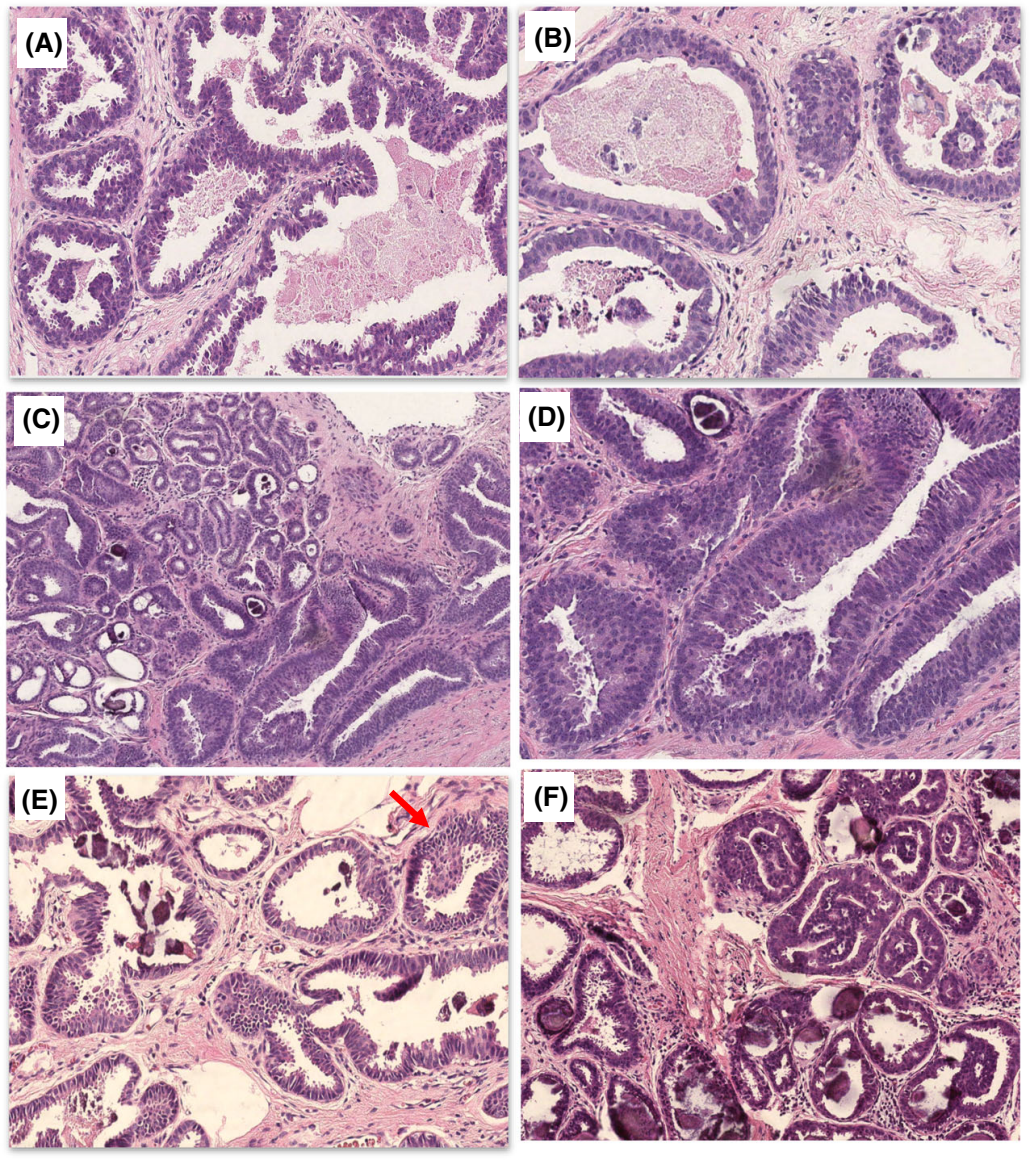
Group 2 cases with > 70% agreement among participants. Case 2 (**A,B**) showed an area of columnar cell hyperplasia with elongated stratified nuclei and micropapillae with irregular luminal outlines in (**A**), and another area with more atypical rounded nuclei, more rigid outlines, loss of polarity and no apical snouting (**B**). Case 5 (**C,D**) showed calcified columnar cell change and hyperplasia with a more hyperchromatic area at low power in (**C**). The ducts were lined by cells with round and oval basal nuclei containing tiny, but visible, nucleoli and showed stratification and mounds, with no significant architectural complexity (**D**). Case 7 (**E**) was labelled as borderline favouring atypia by most participants. The ducts were irregularly dilated without rigid outlines or a complex architecture except for what some described as a micropapillary structure (arrow). The lining was stratified population with ‘worrying’ nuclear enlargement and loss of polarity (**E**). Case 9 (**F**) shows the contrasting appearances of columnar cell hyperplasia (right) and the atypical area on the left amounting to atypical ductal hyperplasia/atypical intraductal epithelial proliferation. The latter showed fusion of resulting in slit‐like, rather than punched‐out spaces. Nevertheless, the nuclei were rounded and definitely atypical with loss of polarity.

Unifying features of group 2 cases (cases 2, 5, 7, 9; Figure 3) were the involvement of ≤ 2.5 mm or multiple smaller areas, abundant microcalcification and focal or subtle architectural abnormalities; for example, case 2 (Figure 3A,B). In case 5 (Figure 3C,D), the cells were tall columnar, with crowding and stratification. The nuclei were hyperchromatic with visible nucleoli. This suggested FEA with a ‘low‐grade colonic adenoma’ appearance. Cases 7 and 9 (Figure 3E,F) were more akin to AIDEP/ADP due to the architectural atypia. The presence of apical snouts had limited influence on the diagnostic decision as FEA, while increased mitotic activity is often seen in association with marked cytological atypia, denoting the presence of high‐grade clinging or flat‐type DCIS.

#### Group 3 cases (achieving < 70% agreement)

**Figure 4:**
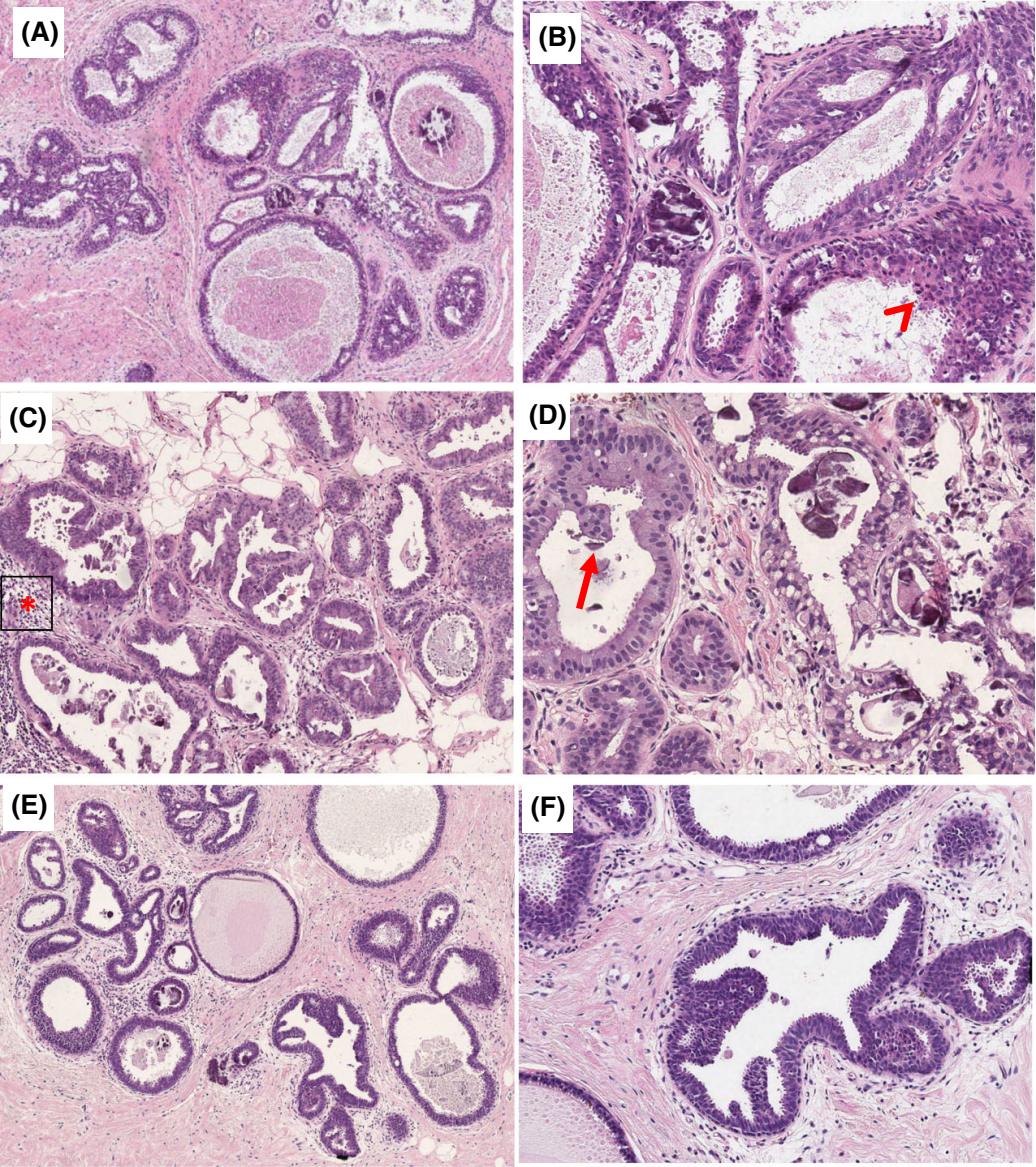
Group 3 cases with the lowest agreement. Case 4 (**A**,**B**) contained focally dilated ducts with rounded and irregular contour and complex architecture in (**A**). Pathologists who favoured atypia focused upon the more rigid spaces (**B**; upper left), the loss of polarity and nuclear enlargement being above that expected in low‐grade atypia (**B**; arrowhead). Case 8 (**C,D**) showed irregular outlines of terminal duct lobular units (TDLUs) with stratification and calcification. The cells were cuboidal to columnar cells with rounded to oval nuclei and moderate amounts of cytoplasm (**C**). In (**D**) focal intracytoplasmic vacuoles with loss of polarity were present (**D**; left). Pathologists who favoured atypia noted early micropapillae (arrow), nuclear enlargement and more prominent chronic inflammation (focally intralobular; marked with asterisk). Other pathologists considered the possibility or early apocrine change, and suggested that the abundant cytoplasm, lack of evenly spaced monomorphic nuclei and lack of architecture atypia are sufficient evidence for their non‐atypical diagnosis. Case 10 (**E,F**) showed dilatation of terminal duct lobular units (TDLUs), which is predominantly rounded with complex architecture in the form of micropapillae‐like excrescences and bridges. Pathologists who called this atypical were concerned about the excrescences, which they viewed as true micropapillae, together with the notable loss of polarity and nuclear enlargement, variation in nuclear size and roundedness. Other pathologists noted that the features do not amount to true micropapillae and would fit with columnar cell proliferation, with reassuring nuclear overlapping; overall falling short of either flat epithelial atypia (FEA) or atypical ductal hyperplasia (ADH).

All these cases lacked the rounded regular nuclei of FEA and showed mounds, at the most. The presence of micropapillary‐like structures was a cause for disagreement regarding whether they indicate atypia (true micropapillae) or were within the range of CCH (pronounced epithelial tufts and gynaecomastoid type changes). Roman bridges and rigid cribriform spaces were absent (cases 4, 8, 10; Figure 4). Cases 4 and 10 showed disorganisation and a mixed ‘hyperplastic’ appearance with complex architecture. Some participants suggested that the nuclei were more akin to intermediate‐grade atypia, while others did not consider the nuclei atypical, but a form of florid CCH with stratification. Assessment of polarity was difficult in the context of epithelial stratification and tufting, particularly with irregular rather than monomorphic nuclei. Furthermore, loss of polarity was used by participants as a criterion of atypia, and as an indication for the lack of atypia by other participants. Terminal duct lobular units (TDLUs) distension by ‘blue’ mucin in case 8 was described as worrying by some participants, which was suggested by a previous study.^26^

**Figure 5:**
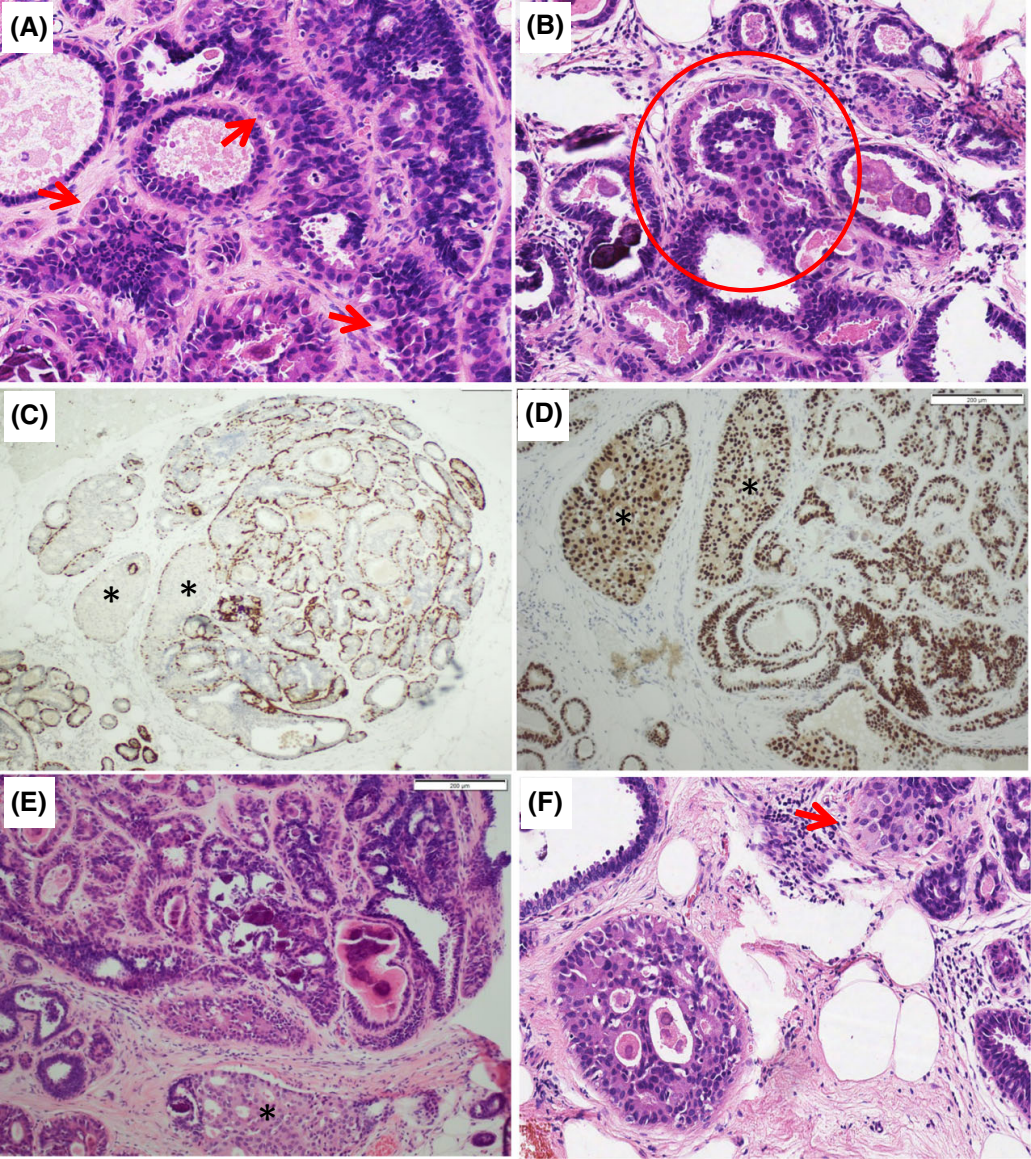
Case 1 before and after immunohistochemistry. (**A,B**) The original haematoxylin and eosin (H&E) sections showed columnar cell change with typical snouting and calcification, admixed with larger cells basal that had more prominent eosinophilic cytoplasm (**A**, arrow). These formed small luminal aggregates in one duct (**B**, circle), but no punched‐out spaces or bridges. (**C,D**) Immunohistochemistry for the basal markers (CK5/p63) and oestrogen receptor (ER), respectively, showed a group of dilated ducts (*) that lack an admixed basal component within the luminal proliferation, and strong homogeneous nuclear expression of ER, which was also observed in the background columnar cell change. (**E, F**) H&E recuts after immunohistochemistry showed that the luminal proliferation (bottom) consists of larger cells similar to those seen in **A**, with poorly formed punched‐out spaces and pagetoid spread in neighbouring lobules (**F**, arrow).

Case 1 (Figure 5) showed columnar cells admixed with larger cells with abundant eosinophilic cytoplasm. When discussed at the consensus meeting, it was agreed that haematoxylin and eosin diagnosis would be used in the analysis, but IHC would be performed to elucidate the nature of these cells, with the intention of excluding the case if they represent lobular neoplasia. Surprisingly, IHC sections revealed a monomorphic neoplastic population of epithelial cells, with complex architecture and pagetoid‐type spread into adjacent lobules. On rediscussion, the participants agreed that this case was AIDEP, but falls short of an upgrade to DCIS. This case demonstrates the value of examining deeper levels and IHC in borderline atypical cases.

**Figure 6:**
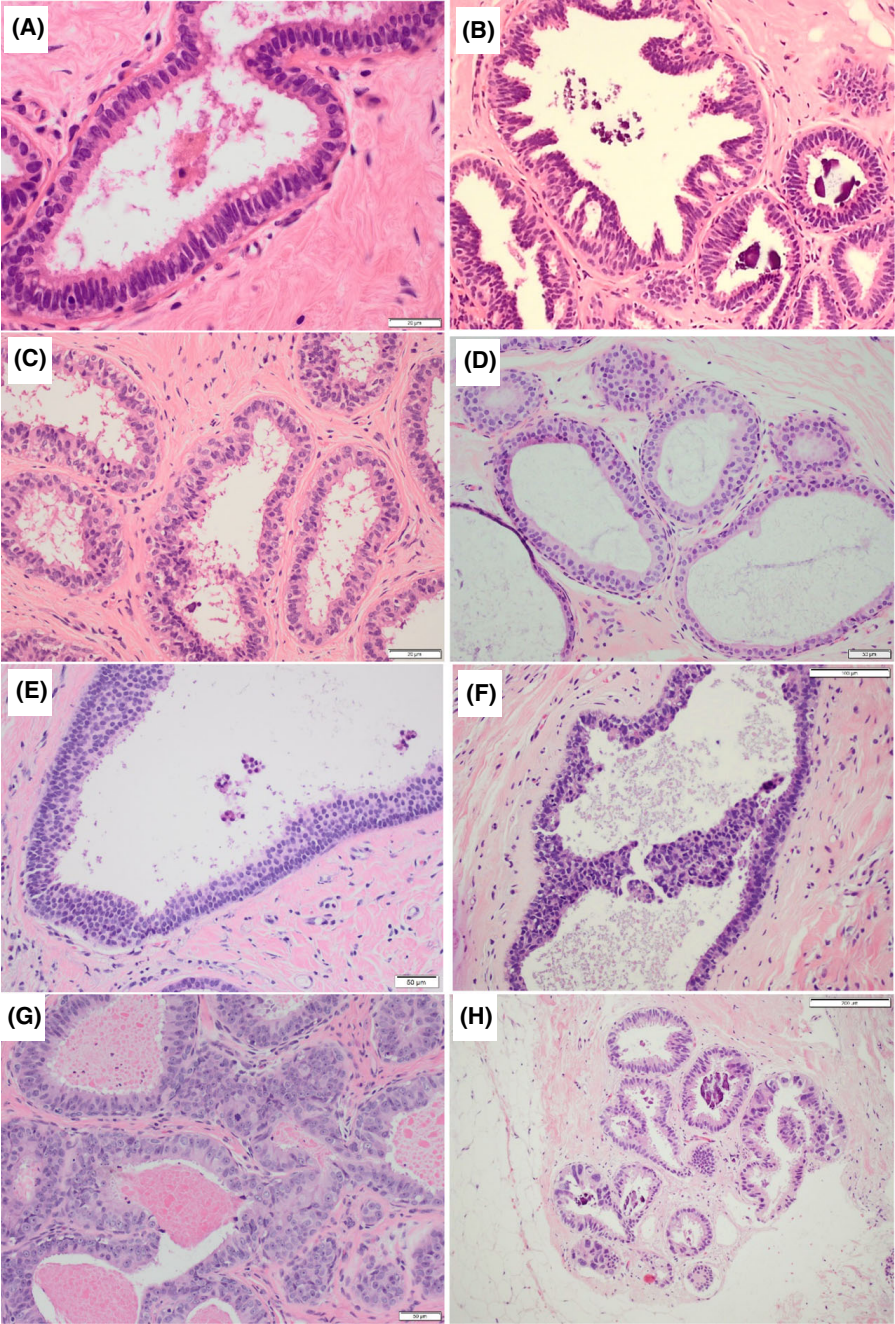
Examples of a range of columnar cell lesions that are fairly straightforward to classify. (**A**) Columnar cell lesions (CCC) composed of a single layer of columnar cells with elongated parallel and compressed nuclei that have smooth chromatin and inconspicuous nucleoli. Mild overlapping is seen and nuclear snouts present. There is no nuclear rounding or irregularity and no stratification. (**B**) Columnar cell hyperplasia (CCH) shows stratification of columnar cells with gradual nuclear crowding and overlapping. The stacked columnar cells project into the lumen, leading to tufts, mounds and simple micropapillae. (**C**) CCH in which there is some loss of polarity and a lack of parallel orientation perpendicular to the basement membrane. The nuclei are more oval than rounded with some irregularity and the chromatin is smooth, no conspicuous nucleoli. The myoepithelial cells are also prominent. (**D,E**) Two examples of flat epithelial atypia (FEA). (**D**) FEA as classically described consists of rounded rigidly dilated terminal duct lobular units (TDLUs) lined by one to several layers of mildly atypical monotonous cuboidal to cells, with round, uniform and enlarged nuclei with mild hyperchromasia, increased nuclear‐to‐cytoplasmic ratio and sometimes prominent nucleoli. They lack overlap and are often central with loss of the perpendicular orientation to the basement membrane. (**E**) A variant morphology of FEA showing similar features but with relatively small clonal cells that have scanty delicate cytoplasm and small regular rounded nuclei. (**F**) The cuboidal cells lining this rigidly dilated duct are also small and have relatively irregular overlapping nuclei with smooth chromatin. There is stratification, marked loss of polarity and a suggestion of bar/early bridge formation. This degree of architectural atypia makes the diagnosis of atypical ductal hyperplasia/atypical intraductal epithelial proliferation (AIDEP/ADH) more appropriate. (**G**) These mildly dilated ducts are lined by moderately pleomorphic and mitotically active cells that have dense eosinophilic cytoplasm and enlarged oval vesicular nuclei with conspicuous small nucleoli. The lumina contain inspissated eosinophilic secretion, with pyknotic debris (arrow, left above). (**H**) Mildly dilated lobules with luminal microcalcification lined by a single layer of columnar cells that have variable appearances. In most areas, the nuclei are small elongated and oval, and regularly arranged perpendicular to the basement membrane. However, there are admixed high‐grade cells with enlarged nuclei, with irregular outlines, overlapping and focal binucleation. Nucleoli are not conspicuous.

**Figure 7:**
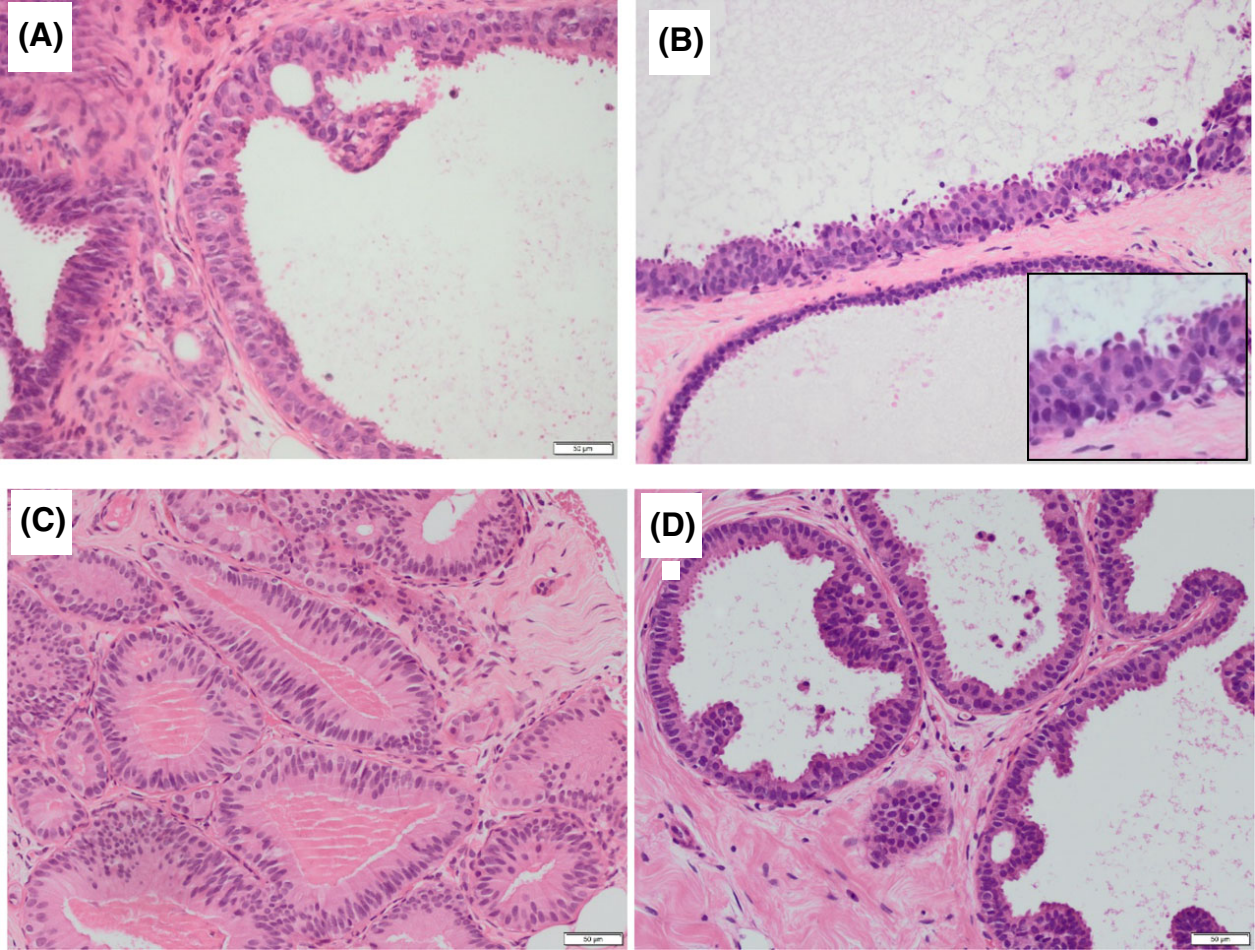
Examples of columnar cell lesions that are difficult to classify. (**A**) Opinion split between atypical and non‐atypical; (**B–D**) majority favouring atypia) (**A**) A dilated duct lined by small stratified cells with disorganisation and nuclear overlapping, is present adjacent to ducts that show columnar cell hyperplasia. There is an early punched‐out space, above which the bridging cells form an angled but tapering bar. The nuclei are vesicular with mildly irregular outlines, occasional pinpoint nucleoli. (**B**) Two rigidly dilated ducts, one is lined by a single layer of small cuboidal cells with rounded nuclei (bottom) and the other is lined by cells with larger, still round, overlapping nuclei, with some disorganisation and loss of polarity (inset). There are snouts and early mounds but no micropapillary structures, bars or bridges. The majority view was that this would be considered atypical (B3 on a core biopsy). (**C**) Duct spaces and lobules lined by columnar cells that show marked stratification without significant overlap. The nuclei are oval or rounded and have a smooth chromatin and nuclear outlines with inconspicuous nucleoli. The cytoplasm is abundant and densely eosinophilic. The luminal border lacks snouting and there is dense luminal eosinophilic material without nuclear debris. (**D**) Columnar cells with oval and rounded nuclei that have smooth chromatin, smooth nuclear outlines and inconspicuous nucleoli. They show mild architectural changes in the form of focal mounds, bridges and early bars, without background stratification.

Finally, based on the controversial areas identified in the first set of cases, a photomontage includes a range of CCLs that bring into focus the areas of difficulty and how to safely assess them. We provide this as a visual aid for practising pathologists in the diagnosis of FEA and AIDEP arising in a background of CCLs (Figures 6 and 7).

## Discussion

In this study, challenging VABs containing atypia that defied straightforward categorisation into FEA or ADH/AIDEP using established criteria, were reviewed by an experienced panel of breast pathologists to assess the level of diagnostic agreement and identify reasons for disagreement. The cases had comparable age, history and outcome, and the exercise was purely academic with no clinical factors that could influence pathologists’ suspicions. The participants do not normally work together and are based in different regions/cities in the United Kingdom and the Republic of Ireland. The criteria applied were those used in normal daily practice without any ‘adjustments’. There was no attempt to determine who was ‘right’, but how the decisions for the presence or absence of atypia were reached.

Considering that the cases were preselected to represent non‐classic examples of FEA/AIDEP, and that no written diagnostic or training set/tutorial were provided before case review, the low diagnostic agreement in this study is not surprising. Interobserver agreement of FEA was found to be excellent only in a single study that included participants receiving a written summary of the standardised criteria, PowerPoint tutorials and instructions to categorise equivocal cases into the ‘not atypical’ category.^27^

Elston *et al*.^28^ analysed the level of interobserver agreement in the diagnoses of ADH and DCIS among 23 pathologists using a spectrum of these cases and predefined diagnostic criteria, but the kappa indices for ADH were poor (0.35). A recent meta‐analysis of interobserver variation in ADH showed kappa values ranging from poor agreement (kappa = 0.17) to substantial agreement (kappa = 0.69), with higher agreement rates in studies from experienced breast pathologists and/or those who received prior tutorial sessions for atypia diagnosis.^29^

In this study, the presence of rigid punched‐out cribriform spaces, thick Roman bridges and club‐shaped micropapillae (even focally) made diagnostic agreement easier to achieve, but our experienced participants struggled to achieve consensus for the presence or absence of FEA or some ‘other form of atypia’, and gave subjective weighting to the criteria of cytological atypia when architectural atypia was absent. This finding is similar to a previous study, which attributed lack of consensus for the diagnosis of atypia in 4% of their cohort due to borderline features and abortive micropapillae.^30^ Higher agreement was noted if the changes were more widespread, but some participants considered the atypical population not ‘clonal enough’ if many lobules were involved.

Our study demonstrated the value of group review and discussion in improving interobserver agreement in this subset of difficult to classify CCLs with atypia. It also showed that some cases fail to achieve a consensus diagnosis, despite lengthy and detailed analysis and discussion. Reducing interobserver variation can also be addressed by the development of more robust diagnostic criteria, clearer cut‐offs, additional educational efforts, the use of digital pathology and artificial intelligence (AI). Image analysis could potentially provide better standardisation in the diagnosis of these difficult lesions, provided that interobserver variability is not introduced in these deep‐structured machine‐learning models.

There is no reliable estimate of the prevalence of controversial breast atypia cases similar to those presented here in routine practice, and they are rarely discussed or described in the literature. They represented 4% of cases in one study^30^ and 16% in another.^20^

Limitations of the study include the lack of a gold standard (ground truth/reference/right diagnosis) on which assessments and opinions could be ranked, and the absence of differential outcome data that could, in retrospect, separate atypical and non‐atypical cases. Another limitation is that diagnosis was based on a single breast biopsy slide in a research setting without additional IHC and/or extra levels.

CCLs with low‐grade atypia that do not fulfil the criteria of FEA are a pitfall and point of disagreement among breast pathologists, some of whom consider low‐grade atypia that falls outside FEA to be within the acceptable range of proliferative breast change. Our study shows that most expert UK breast pathologists do not dismiss cytological atypia that does not morphologically resemble FEA.

A review of the current diagnostic criteria and provision of clearer guidance that can be objectively applied such as a size cut‐off (even if arbitrary), or giving weighted importance to the nuclear and architectural diagnostic criteria, may reduce interobserver disagreement. Alternatively, it may be helpful to introduce an ‘atypical CCLs’ category to encompass lesions with convincing cytological atypia, but where the classical criteria of FEA do not fit. In the presence of accompanying architectural atypia, the term AIDEP/ADH should still apply, and we do not propose any change to the use of the latter terms, whether or not arising within an atypical CCL.

In terms of management, if the only abnormality present is FEA or AIDEP, the management is VAE, not surgery, in the United Kingdom. Therefore, if the term ‘atypical CLL’ is adopted, there will be no clinical impact on management. In other parts of the world where surgical excision is offered, we stress that ‘atypical CCL’ is an alternative to FEA and should be managed as FEA, not as AIDEP/ADH.

Studies that define the range of atypical features in CCLs would be helpful to standardise their diagnosis, resolve interobserver disagreements in challenging cases, reduce unnecessary VAEs, improve recruitment to clinical trials and reduce the highly variable outcome data recorded for atypical breast lesions nationally and internationally.

## Conflicts of interest

The authors declare that they have no conflicting/competing interests.

## Data Availability

Slides analysed in the first part of this study are available online at https://www.virtualpathology.leeds.ac.uk/slides/browser/?path=%2FResearch_4%2FTeaching%2FEducation%2Froyal_Free_london%2FQA%2F2021_02_09. Data sets analysed during the current study, other than those that are included in this published article (its supplementary information files), are available from the corresponding author upon reasonable request.
